# What Drives the Trickle-Down Effect of Calling Orientation From Supervisors to Subordinates? The Perspective of Social Learning Theory

**DOI:** 10.3389/fpsyg.2019.00905

**Published:** 2019-05-09

**Authors:** Baoguo Xie, Wenxia Zhou, De Xia, Yongxing Guo

**Affiliations:** ^1^School of Management, Wuhan University of Technology, Wuhan, China; ^2^School of Labor and Human Resources, Renmin University of China, Beijing, China; ^3^DiggMind Psychometric Testing Technology Co., Guangzhou, China

**Keywords:** trickle-down, social learning theory, calling orientation, role modeling, organizational status

## Abstract

Despite an increase in research on calling orientation, few studies have investigated its antecedents. Drawing on social learning theory, we hypothesized that subordinates’ perceptions of their supervisor’s role modeling mediate the relationship between supervisor’s and subordinates’ calling orientations. Supervisor’s organizational status is supposed to augment the trickle-down process for calling orientation. We used multilevel modeling to test these hypotheses in a sample of 738 subordinates nested in 77 work teams in Chinese firm. We found that supervisor’s calling orientation was positively related to subordinate’s calling orientation and that the relationship was fully mediated by subordinates’ perceptions of role modeling. Additionally, the relationship between supervisor’s calling orientation and subordinates’ calling orientation via role modeling was moderated by supervisor’s organizational status at the second stage.

## Introduction

Work orientation, or the viewing of one’s work as a job, or career, or calling explains the different meanings that people find in their work. A calling can provide people with a deep sense of meaning (Hall and Chandler, 2005; Weir, 2013) and so interest in callings has increased over the last two decades. In the academic literature, many studies illustrated that a sense of calling is beneficial for employees themselves and their employing organizations. Empirical studies have found that employees with a calling report greater psychological and subjective career success (Praskova et al., 2014; Xie et al., 2016), well-being (Duffy and Dik, 2013; Duffy et al., 2017), in- role (Park et al., 2016; Lee et al., 2018) and extra-role performance (Park et al., 2016; Xie et al., 2017; Lee et al., 2018).

Given that having a calling has benefits for the individual concerned and for their organization there is both theoretical and practical interest in ways of developing an individual’s calling orientation (Duffy et al., 2011; Longman et al., 2011; Dobrow, 2013; Bott and Duffy, 2015; Haney-Loehlein et al., 2015; Creed et al., 2016; Esteves and Lopes, 2017). Based on the results of a grounded theory study Longman et al. (2011) argued that calling orientation was shaped by the contextual factors such as theological influences, family realities, cultural expectations and life circumstances. The first empirical investigation of predictors of calling was carried out by Duffy et al. (2011) in a sample of medical students. They found that life meaning positively predicted calling 2 years later. A longitudinal study (Dobrow, 2013) of musicians indicated that individuals who were more behaviorally involved in music and gained greater social comfort from music experienced a higher sense of calling. Recently Esteves and Lopes (2017) found that nurses and nursing assistants who received challenging job demands experienced a higher sense of calling.

Although a few recent studies have investigated the antecedents of calling orientation empirically (e.g., Duffy et al., 2011; Dobrow, 2013; Bott and Duffy, 2015; Creed et al., 2016; Esteves and Lopes, 2017) the predictors of calling orientation are still poorly understood. One weakness in the calling literature is that previous empirical research focused exclusively on individual predictors of calling orientation, neglecting the contextual factors that also contribute to the development of calling orientation (Hall and Chandler, 2005; Longman et al., 2011). The relational theory of working (Blustein, 2011) posits that working is embedded in external and internal relational contexts. Supervisors shape their subordinates’ identity, identification (Sluss and Ashforth, 2007) and sense of meaning (Pratt and Ashforth, 2003; Rosso et al., 2010). It is likely, therefore, that they also can shape subordinates’ calling orientation. “Callings involve work that is rooted in people’s values” (Schabram and Maitlis, 2017, p. 584) and values can be transmitted from supervisor to subordinate. Unfortunately, there has so far been no investigation of the supervisor’s role in development of individuals’ calling orientation.

This study addressed this issue and extends prior calling research in several ways. First, as supervisors shape their subordinates’ identity, identification, and sense of meaning in the workplace (Pratt and Ashforth, 2003; Sluss and Ashforth, 2007; Rosso et al., 2010; Carton, 2018), we examined the supervisor’s role in the development of supervisors’ calling orientation. Second, drawing on social learning theory (Bandura, 1977, 1986) we examined subordinates’ perceptions of role modeling of their own supervisor and supervisor’s organizational status as potential explanatory mechanisms for the relationship between the supervisor’s and subordinate’s calling orientation. Third, in line with the new interest in the trickle-down models that link supervisors’ perceptions, attitudes and behaviors to similar constructs at the subordinate level, we investigated the trickle-down effects of calling orientation. In doing so we not only examined the contextual predictors of calling orientation but also demonstrated another form of trickle-down in the workplace.

### Theoretical Background and Hypothesis Development

#### Conceptualization of Calling Orientation

According to the neoclassical callings, calling orientation is broadly defined as “transcendent summons, experienced as originating beyond the self, to approach a particular life-role in a manner oriented toward demonstrating or deriving a sense of purpose or meaningfulness and that holds other-oriented values and goals as primary sources of motivation” (Dik and Duffy, 2009, p. 427), and characterized by transcendent source/destiny, purposeful/meaningful, and prosocial motivations (Dik and Shimizu, 2019). Transcendent source/destiny is characterized by a sense called by “an external or transcendent caller (e.g., God, salient social needs, a family legacy) or a sense of destiny” (p. 3). Purposeful/meaningful refers to “a meaningful and purposeful approach to work” (p. 3). Prosocial motivation is that the individual is “motivated by a prosocial desire to use his or her gifts toward positive societal impact” (p. 3). The neoclassical perspective of calling has been supported by a qualitative study (Zhang et al., 2015a) and a measurement study (Zhang et al., 2015b) in Chinese context.

#### Trickle-Down Effects

In the organizational psychology literature, the trickle-down model is often used to link supervisors’ perceptions, attitudes, and behaviors to similar constructs at the subordinate level. The assumption of trickle-down models is that subordinates are susceptible to their leader’s experiences. Masterson (2001) firstly examined the trickle-down effects of organizational justice. Since then, a great number of studies investigated a broad range of trickle-down phenomena including justice perceptions, positive/negative affect, organizational identification, psychological capital, psychological distress, work engagement, and so on (see review, Wang et al., 2015; Li et al., 2016; Lu et al., 2018). In terms of theoretical explanation, the social learning theory is predominantly used as an explanatory theory to understand trickle-down effects (e.g., Brown et al., 2005; Simons et al., 2007; Mawritz et al., 2012; Ambrose et al., 2013; Lu et al., 2018).

#### Hypothesis Development

**FIGURE 1:**
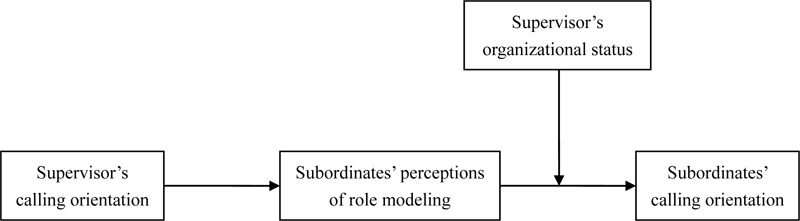
The proposed research model.

Drawing on social learning theory we hypothesized that: (a) supervisor’s calling orientation is positively related to subordinates’ calling orientation, (b) subordinates’ perceptions of role modeling mediates this relationship, and (c) a supervisor’s organizational status augments the indirect effect of his or her calling orientation on subordinates’ calling orientation through subordinates’ perceptions of role modeling. The research model is shown in Figure 1.

##### Calling orientation of supervisors and subordinates

Social learning theory states that learning is a cognitive process that occurs in a social context and that learning can happen purely on account of observation or instruction (Bandura, 1977, 1986). According to social learning theory there are at least two reasons why supervisors’ calling orientation should be positively associated with their subordinates’ calling orientation. First, supervisors having a calling often have a clear sense of purpose and personal mission (Elangovan et al., 2010; Park et al., 2018) that drives them to engage in sense-making activities and express their views on work issues when they interact with team members. Supervisors’ visibility and influence means that their subordinates’ perceptions of the meaning of their work will be susceptible to supervisors’ sense-making activities (Bartunek et al., 1999; Maitlis, 2005; Zhang and Bartol, 2010). In social learning theory terms sense-making can be viewed as a kind of direct instruction that changes the meaning of work (Carton, 2018). Thus, a supervisor with a calling can transmit calling orientation to his or her direct subordinates by sense-making. Second, the construct of calling encompasses an observable pattern of behavior as well as a specific self-perception (Dik et al., 2012); in other words, people can detect whether someone has a calling. In a scale development study Dik et al. (2012) found that self- and informant-reported calling scale scores were correlated. Based on this theoretical work and empirical evidence, we argue that subordinates can also observe a supervisor’s calling orientation in team contexts and will unconsciously or consciously mimic the behaviors that signal the leader’s calling orientation. Hence,

***Hypothesis 1:*** *The supervisor’s calling orientation is positively associated with his or her subordinates’ calling orientation*.

##### The mediating role of subordinates’ perceptions of role modeling

Role modeling is a key construct in social learning theory and is the psychological mechanism underlying learning in social contexts (Bandura, 1977, 1986). “Role models are typically people who have power, competence, and higher levels of status, and for whom behavioral information is available” (Wo et al., 2015, p. 1852). Subordinates’ perceptions of role modeling of their own supervisor reflect the extent to which a subordinate relies on his or her supervisor as a source of information about appropriate behaviors, and “covers a broad range of psychological matching processes, including observational learning, imitation, and identification” (Brown et al., 2005, p. 119). Supervisors have higher status than those at lower organizational levels and formal authority over them (Yukl and Lepsinger, 2004) so they are naturally seen as legitimate sources of legitimate information (Ambrose et al., 2013) and are the targets of observational learning (Mayer et al., 2012). Thus, according to social learning theory, the perceptions of role modeling should mediate the positive relationship between supervisor’s and subordinates’ calling orientation.

***Hypothesis 2:*** *Role modeling mediates the positive relationship between supervisor’s calling orientation and subordinates’ calling orientation*.

##### The moderating role of supervisor’s organizational status

In this study we use the term supervisory status to refer to subordinates’ perception of the informal organizational status of a supervisor. Followers’ judgment of their supervisor’s informal organizational status if often based on “(a) the organization’s positive valuation of the supervisor and care about the supervisor’s welfare, (b) the supervisor’s influence over important organizational decisions, and (c) the authority and autonomy allotted supervisors to carry out their job responsibilities” (Eisenberger et al., 2002, pp 567–568). Supervisory status represents subordinates’ perception of their supervisor’s authority and power in the organization. Social learning theory suggests that high standing in power and authority contribute to modeling effectiveness (Bandura, 1977, 1986). Experimental studies (Bandura et al., 1963; Bussey and Bandura, 1984; Goldstein, 1986) also showed that observers were more likely to adopt or learn behavior if the actor modeling the behavior was of high social status. Thus, according to social learning theory, subordinates are more likely to adopt or learn attitudes and behaviors from their supervisor if he or she has high organizational status. Hence hypothesis 3:

***Hypothesis 3:*** *The supervisor’s organizational status moderates the positive indirect effect of his or her calling orientation on the calling orientation of subordinates via role modeling, such that the indirect effect is stronger if the supervisor’s organizational status is high*.

Dik et al. (2012) argued that calling reflects not just self-perception, but also an observable pattern, so others can detect whether or not an individual has a calling. If one can observe whether someone has a calling there should be a correlation between self- and informant-reports of calling. Dik et al. (2012) found that such a correlation existed in a sample of college students. This study attempts to replicate this result in working adults to demonstrate its generality.

***Research Question:*** *Can supervisors’ calling orientation be observed by their subordinates?*

## Materials and Methods

This study was carried out in accordance with the recommendations of Wuhan University of Technology Institutional Review Board. The protocol was approved by the Wuhan University of Technology Institutional Review Board. All subjects gave written informed consent in accordance with the Declaration of Helsinki.

### Procedure and Participants

The current dataset is part of a large-scale data collection on calling orientation (Xie et al., 2016; Appendix). We recruited the participants from a bank located in South China. With the help of the human resources director, 1355 employees belonging to 121 work teams were targeted, and the 1355 employees and 121 supervisors (middle managers) were invited to participate in our survey through an e-mail over the organization’s intranet.

To incentive the participants, we promised that their responses would be confidential and they would get feedback about their own assessment report if they wanted. To match employees to their respective teams and supervisor, an anonymous team number was given to employees and supervisors. In total, 1355 questionnaires and 121 questionnaires were distributed to employees and supervisors, respectively. We received 1026 responses from employees and 107 response from supervisors. Following Klein et al. (2001) recommendation, we excluded teams with fewer than three respondents. Furthermore, we excluded teams whose supervisors had worked with the members for less than a year (Lu et al., 2018). Following this procedure and matching the responses of supervisors to those of their subordinates, we finally obtained a valid sample of 77 supervisors and 738 subordinates from 77 teams. On average, there was 9.58 subordinates in a work team. Among the supervisor sample, their average age was 41.81 years old (*SD* = 6.51), average organizational tenure was 11.25 years (*SD* = 4.75), 50.65% were female, and 79.22% of them held at least a bachelor degree. Among the subordinate sample, their average age was 37.75 years old (*SD* = 5.70), the average organizational tenure was 5.24 years (*SD* = 4.20), 67.28% were female, and 84.76% of them held at least a bachelor degree.

### Instruments

Supervisor’s calling orientation was rated by the supervisor concerned and by his or her subordinates. Supervisor’s organizational status was rated by direct subordinates and analyzed as the aggregate of these scores. Subordinates’ calling orientation and perceptions of their supervisor’s role modeling were reported by subordinates. Common method bias can be minimized by collecting data from multiple sources (Podsakoff et al., 2003).

#### Calling Orientation of Supervisors and Subordinates

Calling orientation was measured with the 12-item Calling and Vocation Questionnaire (CVQ; Dik et al., 2012). Responses were given on a six-point Likert scale ranging from 1 (*not at all true of me*) to 6 (*totally true of me*). A sample item is “I am pursuing my current line of work because I believe I have been called to do so.” In the present study, the Cronbach’s α for calling orientation among supervisors is 0.93 and that for calling orientation among subordinates is 0.90. Subordinates also rated their supervisor’s calling orientation using the two-item Brief Calling Scale (BCS; Dik et al., 2012). The BCS was adapted to suit the current research context. The two items were “My leader has a calling to a particular kind of work” and “I have a good understanding of my leader’s calling as it applies to his or her career.” Subordinates were asked to indicate their agreement on a scale ranging from 1 (*totally disagree*) to 6 (t*otally agree*). In our sample Cronbach’s α for the BSC was 0.72.

#### Subordinates’ Perceptions of Role Modeling

A four-item scale was used to assess the extent to which subordinates adopted the role modeled by their supervisor (Rich, 1997). The four items were “My supervisor provides a good model for me to follow,” “My supervisor leads by example,” “My supervisor sets a positive example for me to follow,” and “My supervisor acts as a role model for me.” Subordinates were asked to indicate their agreement with each item using a six-point Likert scale (ranging from 1 = *totally disagree* to 6 = *totally agree*). Cronbach’s α for the four items was 0.93.

#### Supervisor’s Organizational Status

In Eisenberger et al.’s (2002) study, a unidimensional scale with 12 items was developed to assess the supervisor’s organizational status. Consistent with the employees’ judgments, the scale measures the supervisor’s informal status in the organization from value, influence, and autonomy. In the present study, we selected four high-loading items from Eisenberger et al.’s (2002) scale to assess the supervisor’s informal status in the organization. The items selected were “The organization holds my supervisor in high regard” (value), “The organization gives my supervisor the chance to make important decisions” (influence), “The organization values my supervisor’s contributions” (value), and “The organization gives my supervisor the authority to try new things” (autonomy).

Because the shared unit property approach (Kozlowski and Klein, 2000) was adopted to collect data, we conducted multilevel confirmatory factor analysis (MCFA) (Dyer et al., 2005) and calculated aggregation indices of supervisor’s organizational status (Chan, 1998). The result of MCFA showed that there was a good unidimensional structure at both within-level and between-level (χ^2^ = 33.32, *df* = 4, *p* < 0.001, CFI = 0.97, RMSEA = 0.10, SRMR _between_ = 0.02, SRMR _within_ = 0.02), and standardized factor loadings ranged from 0.63 to 0.86 at within level and from 0.98 to 0.99 at between level. The aggregation indices of supervisor organizational status [*r*_wg_ = 0.91, ICC (1) = 0.13, ICC (2) = 0.91] exceeded the conventional standards of agreement [*r*_wg_ ≥ 0.70, ICC (1) ≥ 0.12, ICC (2) ≥ 0.70] (James, 1982; Liao and Zhuang, 2012). Taken together, it was support for aggregation of the supervisory status measure to a group level construct. The Cronbach’s α was 0.87.

#### Control Variables

Prior studies have shown that age, organizational tenure, gender, and educational level could influence calling orientation (Wrzesniewski et al., 1997; Duffy and Sedlacek, 2007; Dobrow, 2013) so the age, organizational tenure, gender (0 = *male*; 1 = *female*), and educational level (0 = *associate*; 1 = *bachelor*; 2 = *master and above*) of supervisors and subordinates were considered as potential control variables.

## Results

### Preliminary Analyses

The normality of the five variables of interest was first inspected by examining coefficients. Inspection revealed that supervisors’ calling orientation rated by themselves and role modeling had kurtosis levels over one. Thus, following the practice of Sánchez-Oliva et al. (2017), “all models were estimated using the robust maximum likelihood (MLR) estimator available in Mplus 7.4 (Muthén et al., 1998–2015), which provides standard errors and fit indices that are robust to non-normality” (p. 178).

In the present study, we used the same scale (i.e., CVQ) to collect data of the supervisor’s and followers’ calling orientation. Following Vandenberg and Lance’s (2000) approach, we second conducted MGCFA with Mplus 7.4 to test measurement equivalence for CVQ across supervisors and followers. The results were as follows: (a) for configural invariance model, goodness-of-fit indices were χ^2^ = 409.42, *df* = 82, CFI = 0.97, TLI = 0.96, RMSEA = 0.10, SRMR = 0.04, (b) for metric invariance model, goodness-of-fit indices were χ^2^ = 421.18, *df* = 90, CFI = 0.97, TLI = 0.96; RMSEA = 0.09, SRMR = 0.04, (c) for scalar invariance model, goodness-of-fit indices were χ^2^ = 451.98, *df* = 96, CFI = 0.97, TLI = 0.96, RMSEA = 0.10, SRMR = 0.05, and (d) for invariant uniquenesses model, goodness-of-fit indices were χ^2^ = 513.06, *df* = 107, CFI = 0.96, TLI = 0.96, RMSEA = 0.10, SRMR = 0.05. Our model comparisons showed that changes in CFI varied from -0.01 to 0.00. Changes in CFI of -0.01 or less indicate that the invariance hypothesis should not be rejected (Vandenberg and Lance, 2000; Cheung and Rensvold, 2002). Therefore, the strict invariance model (i.e., invariant uniquenesses model) was accepted, which demonstrated that there was measurement variance for CVQ across supervisors and followers.

To justify the appropriateness of multilevel analyses, we next examined the between-group variance in the follower calling orientation. Specifically, the null model with no predictors and the follower calling orientation as the dependent variable was examined. The result showed that between-group variance in the follower calling orientation was significant (*τ*_00_ = 0.03, *p* < 0.01), indicating that there was a group effect and that multilevel analyses was appropriate (Liao and Zhuang, 2012).

As recommended by Bernerth et al. (2018) we finally analyzed whether it was necessary to control eight socio-demographic variables. “By eliminating control variables uncorrelated with the dependent variables we avoided potential spurious effects that controls may have when they are significantly related to the predictor, but not the criterion variables (i.e., we decrease Type I error)” (Kraimer et al., 2011, p. 493). The results of multilevel path analyses with Mplus 7.4 showed that only supervisor’s and subordinate’s organizational tenure significantly predicted subordinate’s calling orientation, so we only controlled for organizational tenure in the hypothesis testing.

### Descriptive Statistics and Correlations

Table 1 shows the means and standard deviations of study variables and the level one and level two correlations between them. As shown in Table 1, the respondents reported moderate to high levels of calling orientation and supervisors (*M* = 4.97, *SD* = 0.72) reported higher levels of calling orientation than subordinates (*M* = 4.50, *SD* = 0.62).

**Table 1:** Descriptive statistics and inter-correlations among variables of interest.

	*M*	*SD*	1	2	3
**Level two**					
(1) Self-reported supervisor calling orientation	4.97	0.72	NA		
(2) Informant-reported supervisor calling orientation	4.60	0.34	0.39^∗∗∗^	NA	
(3) Informant-reported supervisor organizational status	4.51	0.36	0.40^∗∗∗^	0.77^∗∗∗^	NA
**Level one**					
(1) Self-reported subordinate calling orientation	4. 50	0.62	NA		
(2) Self-reported subordinate role modeling	4.64	0.88	0.24^∗∗∗^	NA	

*N = 738 at level one; *N =* 77 at level two; ^∗∗∗^*p* < 0.001.*

### Hypothesis Testing

**FIGURE 2:**
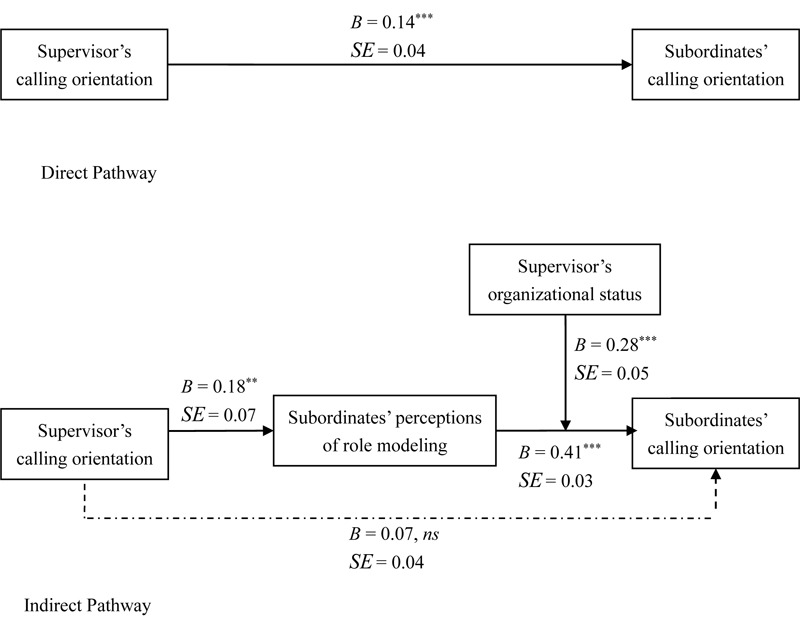
Results of multilevel structural equation modeling assessment (MSEM). ”Unstandardized path coefficients were reported; ^∗∗^*p* < 0.01, ^∗∗∗^*p* < 0.001.

We carried out multilevel structural equation modeling (MSEM) focused on directional relationships between observed variables (Hoyle, 2012). In order to minimize common method bias, self-reported supervisor’s calling orientation was treated as a predictor in testing of the hypotheses. The results are summarized in Figure 2.

Hypothesis 1 was that a supervisor’s calling orientation is positively related to his or her subordinates’ calling orientation. Figure 2 shows that supervisor’s calling orientation was positively associated with subordinates’ calling orientation (*B* = 0.14, *SE* = 0.07, *p* < 0.001). Thus Hypothesis 1 was supported.

Hypothesis 2 was that subordinates’ perception of their supervisor as a role model mediated the relationship between their calling orientation and that of their supervisor. As shown in Figure 2, supervisor’s calling orientation was positively related to subordinates’ perceptions of role modeling (*B* = 0.18, *SE* = 0.07, *p* < 0.001), which was positively associated with subordinates’ calling orientation (*B* = 0.41, *SE* = 0.03, *p* < 0.001). Tofighi and MacKinnon’s (2011) RMediation program was used to compute the 95% confidence interval for the indirect effect. This was [0.02, 0.13], with an average of 0.07 (the ratio of the indirect effect to the total effect = 50%), and did not include 0, thus providing evidence that the association between the supervisor’s calling orientation and the subordinates’ calling orientation was mediated by role modeling.

**FIGURE 3:**
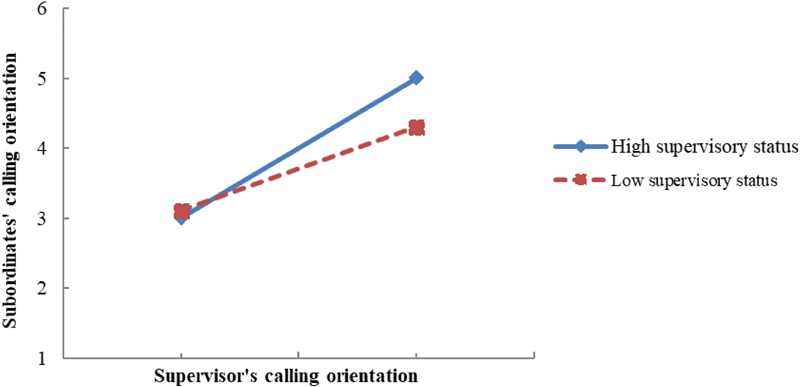
The moderating role of supervisory status on the indirect effect.

Hypothesis 3 stated that supervisory status moderates the indirect positive effect of the supervisor’s calling orientation on his or her subordinates’ calling orientation via subordinates’ perceptions of their supervisor as a role model. This was tested in Mplus 7.4 via moderated mediation analyses with the model constraint command (Liu et al., 2012). Table 2 shows that the indirect effect of supervisor’s calling orientation on subordinates’ calling orientation via role modeling was 0.10 (*p* < 0.001, 95% CI = [0.03, 0.17]) in the context of high supervisory status and 0.06 (*p* < 0.001, 95% CI = [0.02, 0.11]) in the context of low supervisory status. The indirect effect differed significantly at different levels of supervisory status (*B* = 0.04, *p* < 0.001, 95% CI = [0.01, 0.07]). Thus, Hypothesis 3 was supported. To clearly illustrate the moderating role of supervisory status in the indirect effect, Figure 3 was plotted. Figure 3 illustrates that the trickle-down effect of calling orientation via role modeling was more pronounced in the context of high supervisory status than in the context of low supervisory status.

**Table 2:** The results of moderated mediation analyses for Hypotheses 3.

Moderator	Supervisor’s calling orientation→Role modeling→Subordinates’ calling orientation				
	**Stage**		**Effects**		
	**First**	**Second**	**Direct**	**Indirect**	**Total**

Supervisory status					
High (+1 SD)	0.18^∗∗∗^	0.54^∗∗∗^	0.07	0.10^∗∗∗^	0.17^∗∗∗^
Low (-1 SD)	0.18^∗∗∗^	0.34^∗∗∗^	0.07	0.06^∗∗∗^	0.13^∗∗∗^
Difference	0.00	0.20^∗∗∗^	0.00	0.04^∗∗∗^	0.04^∗∗∗^

*+1 *SD* = 0.36 and -1 *SD* = -0.36 for supervisor’s organizational status; ^∗∗∗^*p* < 0.001.*

### Supplementary Analyses

We asked whether a supervisor’s calling orientation can be observed by their subordinates. To answer this question, we asked supervisors’ direct subordinates to rate their supervisor’s calling orientation using the BSC. We calculated interrater reliability, and found that there was high interrater consistency within the teams. The average interrater reliability was 0. 86 (ranging from 0.50 to 1.00, median = 0.89). We also calculated the correlation between subordinate-reported BCS score and supervisors’ self-reported CVQ score. Like Dik et al. (2012; *r* = 0.31), we found a medium-sized (Cohen, 1992) correlation (*r* = 0.39, *p* < 0.001). These results support Dik et al.’s (2012) argument that calling is reflected in observable behavior as well as being a self-perception. Our results also justify the choice of social learning theory as a framework for the research.

## Discussion

The main purpose of this study was to uncover the mechanism of the trickle-down effect of calling orientation from leaders to followers. MSEM showed that calling orientation was transmitted from supervisors to subordinates via subordinates’ perceptions of their supervisor as a role model. Supervisory status played a moderating role in the trickle-down process for calling orientation such that supervisors with high informal status within the organization were more likely to transmit their calling orientation to subordinates through role modeling.

### Theoretical Implications

This study contributes to the calling, trickle-down effects of leadership and social learning theory literature. First, it extends knowledge about antecedents of calling orientation by investigating the effects of supervisor’s calling orientation on subordinate’s calling orientation. “Although the notion of calling can be dated to at least the 16th century, calling as a scientific construct has existed for only a decade or two.” (Xie et al., 2016, pp 75–76), and is in its infancy (Dik and Duffy, 2009). To demonstrate calling’s relative effectiveness there has been a lot of research into the individual and organizational outcomes of calling. The nomological network of a construct also includes its antecedents and so scholars have called for more research on the antecedents of calling orientation (Dik and Duffy, 2009; Duffy and Dik, 2013). There have been a few studies in response to this call (Duffy et al., 2011; Dobrow, 2013; Bott and Duffy, 2015; Creed et al., 2016; Esteves and Lopes, 2017), but the individual and contextual predictors of calling orientation are still poorly understood. This study used social learning theory to provide a framework for an examination of contextual predictors of subordinate’s calling orientation.

Second, this study also extends knowledge about the trickle-down effects of leadership. Recently leadership researchers have shown great interest in models of trickle-down in the organization and found that leaders’ justice perceptions, psychological contracts and breach violations, positive/negative affect, organizational identification, perceived organizational support, leader-member exchange, behavioral integrity, abusive supervision, ethical leadership, psychological capital, psychological distress, and work engagement can transmit from supervisors to subordinates (see review, Wang et al., 2015; Li et al., 2016; Lu et al., 2018). Although leaders can imbue work with meaning by providing employees with a meaningful sense of purpose and mission (Rosso et al., 2010; Carton, 2018), no previous study investigated the trickle-down effects of calling orientation. This study is the first to investigate how leaders shape subordinates’ calling orientation and the extent to which calling orientation trickles down from supervisors to subordinates.

Finally, this study provides direct evidence for social learning theory’s account of trickle-down phenomena. Although social learning theory was considered as the predominant theoretical underpinning of the trickle-down effects of the research phenomena (e.g., Mawritz et al., 2012; Lu et al., 2018) few studies have measured role modeling and examined its role in the trickle-down process directly. An exception is a study by Wo et al. (2015) that measured role modeling and investigated its role in mediating the trickle-down effects of interpersonal justice and informational justice. The study showed that role modeling did not mediate the relationships between a supervisor’s perceptions of interpersonal and informational justice and those of his or her subordinates. Unlike Wo et al. (2015), we found that role modeling accounted for the trickle-down effect of calling orientation. Given that calling is reflected in an observable pattern of behavior (Dik et al., 2012) and has active, behavioral elements (Dik and Duffy, 2009; Elangovan et al., 2010), we believe that social learning theory holds true for explaining the trickle-down process for the constructs including cognitive components as well as behavioral components, such as calling orientation, work engagement, and work passion.

### Practical Implications

The pursuit of meaningful, purposeful work has been encouraged by industry, business leaders, and popular writing (Hurst, 2014; Tian and Wu, 2015). Our results provide some useful practical implications for organizations. To begin, our research demonstrated that calling orientation could spillover from the supervisors to their followers via role modeling. This finding suggests that leaders can develop their followers’ calling orientation by exhibiting calling orientation. In order to increase the levels of employees’ perceptions of their supervisor’s calling orientation, supervisors are first recommended to engage in leader’s sensemaking and sensegiving (Bartunek et al., 1999), through which supervisors can imbue work with meaningfulness by tying employees’ personal goals to a broader mission or purpose. Second, transformational leadership is defined as going “beyond exchanging inducements for desired performance by developing, intellectually stimulating, and inspiring followers to transcend their own self-interests for a higher collective purpose, mission, or vision” (Howell and Avolio, 1993, p. 891). Thus, leaders with a calling are recommended to engage in transformational leadership behaviors including idealized influence, inspirational motivation, intellectual stimulation and individualized consideration. Finally, leaders are recommended to exhibit and express telltale signs of pursuit of a calling through some other behavioral indicators such as passion, active behavioral engagement, self-worth expression in the work roles. Additionally, our results showed that when supervisory status is high, the trickle-down effect of calling orientation was more pronounced than when supervisory status was low. The finding suggests that organizations should favorably view supervisors who have a calling so that they can reap the benefits they could derive from calling orientation. Specifically, organizations need to care about the supervisor’s welfare, and provide them with job autonomy and opportunities for participating in important organizational decisions (Eisenberger et al., 2002).

### Limitations and Future Directions

Our research has several limitations that suggest avenues for further research. First, although the data for this study was collected from supervisors and their direct subordinates to reduce the concern with the common method bias, this study was cross-sectional in nature. Therefore, we should be cautious in causal inferences from our results. Although social learning theory provided theoretical framework that specified the causal order of variables (Mathieu and Taylor, 2006), there may be alternative explanations for our findings. For example, it is possible that leaders who are high in calling orientation recruit followers who are also high in calling orientation. Consequently, a longitudinal research design is required to examine the trickle-down effect of calling orientation from supervisors to followers. Second, to eliminate the potential confounding effects of exogenous variables (i.e., industry characteristics, and organizational culture), the participants from a single organization were recruited. Although this practice can enchance the internal validity of research, it also impairs the generalizability of the results (Kantowitz et al., 2014). Therefore, more research is needed to replicate our findings in other organizational contexts. Third, there are senior or top managers, middle managers, and employees in the organizational hierarchy. The present study only examined the transmission of calling orientation from middle managers to employees. Future research may probe the transmission of calling orientation from top managers, middle managers to employees. Finally, the full range of items assessing the supervisory status was not covered so that the survey short was kept. Although the results of the MCFA and reliability of the revised scale were comparable to those of the original scale (Eisenberger et al., 2002), it is applauded that future studies replicate our findings employing the full scale to measure the supervisor’s informal status in the organization.

## Author Contributions

BX contributed to research idea, data analysis and writing. WZ contributed to research idea and theoretical construction. DX contributed to data analysis and interpretation. YG contributed to data collection and data analysis.

## Conflict of Interest Statement

YG was employed by the company DiggMind Psychometric Testing Technology Co. The authors declare that the research was conducted in the absence of any commercial or financial relationships that could be construed as a potential conflict of interest.

## Appendix

**TABLE A1:** A uniqueness analysis of multiple articles published from the same dataset.

	The current study	Published paper #1
Research question	What are the antecedents of subordinates’ calling orientation.	The effects of subordinates’ calling orientation on their subjective career success and work engagement.
Theories used	Social learning theory (Bandura, 1977, 1986)	Career construction theory (Savickas, 2005)
Constructs/variables	Supervisor’s calling orientation (supervisor-reported at time 1); supervisor’s organizational status (subordinates-reported at time 1); subordinates’ role modeling (subordinates-reported at time 1); subordinates’ calling orientation (subordinates -reported at time 1).	Subordinates’ calling orientation (subordinates-reported at time 1); subordinates’ career adaptability (subordinates-reported at time 1); subordinates’ work engagement (subordinates-reported at time 2); subordinates’ subjective career success (subordinates -reported at time 2).
Unit of analysis	Multilevel	Individual level
Theoretical implications	Calling orientation can transmit from supervisors to subordinates through subordinates’ perceptions of their supervisor’s role modeling, and supervisor’s organizational status facilitates the trickle-down process for calling orientation.	Career adaptability provides an explanatory mechanism for the positive links of calling orientation to work engagement and subjective career success.
Managerial implications	Organizations can shape employees’ calling orientation through two ways: (a) leaders can develop direct followers’ calling orientation by exhibiting calling orientation, and (b) organizations should view favorably leaders having a calling so as to accelerate the benefits they could derive from calling orientation.	(a) Organizations should help employees to connect their work with a tangible, prosocially-oriented purpose to foster employees’ calling orientation, (b) individuals can obtain career meta-competencies through discerning callings, and (c) career counselors could integrate knowledge and skills related to calling into their career interventions to help clients.
